# Comparison of surgical techniques for optimal lead placement in sacral neuromodulation: a cadaver study

**DOI:** 10.1007/s10151-022-02632-x

**Published:** 2022-05-28

**Authors:** C. Dawoud, L. Reissig, C. Müller, M. Jahl, F. Harpain, B. Capek, W. J. Weninger, S. Riss

**Affiliations:** 1 Department of General Surgery, Division of Visceral Surgery, Medical University of Vienna, Vienna, Austria; 2 Division of Anatomy, Medical University of Vienna, Vienna, Austria

**Keywords:** Faecal incontinence, Sacral neuromodulation, Implantation techniques, Anal incontinence, Sacral neurostimulation

## Abstract

**Background:**

Sacral neuromodulation (SNM) is a common treatment for patients with urinary and faecal incontinence. A close contact of the tined lead electrode with the targeted nerve is likely to improve functional outcome.

The aim of this study was to compare the position of the SNM lead in relation to the sacral nerve by comparing different implantation techniques.

**Methods:**

This cadaver study was conducted at the Division of Anatomy of Vienna's Medical University in October 2020. We dissected 10 cadavers after bilateral SNM lead implantation (*n* = 20), using two different standardized implantation techniques. The cadavers were categorized as group A (*n* = 10), representing the conventional guided implantation group and group B (*n* = 10), where SNM implantation was conducted with the novel fluoroscopy-guided “H”-technique. The primary goal was to assess the distance between the sacral nerve and the lead placement.

**Results:**

The electrodes were inserted at a median angle of 58.5° (46–65°) in group A and 60° (50–65°) in group B, without reaching statistical significance. In 8 cadavers, the lead entered the S3 foramen successfully.

The median distance of the lead to the nerve did not show a significant difference between both groups (E0: Group A: 0.0 mm vs. Group B: 0.0 mm, *p* = 0.969; E1: Group A: 0.0 mm vs. Group B: 0.5 mm *p* = 0.754; E2: Group A: 2.5 mm vs. Group B: 2.5 mm *p* = 1.000; E3: Group A: 3.5 mm vs. Group B: 4.0 mm *p* = 0.675). In 2 cases (20%) of the conventional group A, the lead was misplaced and located at the gluteal muscle.

Perforation of the presacral fascia was observed in one lead placement in group A and in two placements in group B.

**Conclusions:**

Both standardized implantation techniques may ensure close electrode proximity to the targeted nerve. Misplacement of the electrode was more often observed with the conventional implantation technique.

## Introduction

Fecal incontinence (FI) is a multifactorial condition affecting up to 20% of the general population [1–3]. The burden of the disease is high, and patient quality of life can be reduced significantly.

The treatment of FI remains challenging, although several new therapies have been introduced in recent years. Sacral neuromodulation (SNM) is commonly used in patients refractory to conservative management. Several clinical studies have confirmed its short- and long-term efficacy [4–6]. Patients need to undergo a test phase before definitive implantation of the generator. This stepwise approach enables a careful selection of appropriate candidates and excludes those who do not respond.

Several factors are considered to contribute to the functional outcome [4, 5]. Fundamental to success is the electrode's localization and its proximity to the sacral nerve [7]. Notably, lead migration was observed in patients with deterioration of initial treatment response, supporting the importance of the exact position of the inserted electrode [8].To date, only a few cadaver studies have evaluated anatomic landmarks for S3 localization and lead placement [9, 10]. Most of them focused on the entry point and the ideal angle for inserting the needle.

Several techniques have been proposed to optimally localize the neural foramina and place the lead close to the sacral nerve root of preferentially S3 [9, 11, 12]. A consensus statement showed that expert opinion varies regarding the ideal surgical approach [1].

A recent expert panel recommended the “H” technique to guide the lead close to the nerve. This standardized technique comprises fluoroscopy-guided marking of S3 by localizing the medial edges of the neural foramina and the inferior end of the sacroiliac joint [13]. In our recent cadaver study, Müller et al. found that this technique led to a close median distance of the electrodes to the nerve [7]. This technique may result in an improved functional outcome and a higher rate of definite implants.

To date, no studies have compared these different surgical techniques.

Therefore, the present cadaver study was performed as a pilot-study to compare the novel “H”-technique to a common non-fluoroscopy guided approach.

## Materials and methods

This study was conducted at the Division of Anatomy of the Medical University of Vienna in October 2020 after approval by the ethics committee (EK #2219/2018).

All individuals had voluntarily donated their bodies to medical education and research and had given their oral and written informed consent.

Body donors with previous urogenital or pelvic surgery and malignant disease affecting the pelvis or sacrum were excluded.

A total of 10 fresh cadavers were included in the study. The median age was 78 years (71–94 years).

Five (3 female, 2 male) were dissected after bilateral SNM implantation as described below [9, 12]. These specimens were categorized as group A, representing the conventional guided implantation group. The other five specimens (3 female, 2 male) (group B) underwent bilateral fluoroscopy-guided lead placement using the “H” technique as previously described by Müller et al. [7]. The results of both implantation techniques were compared (*n* = 20).

Both implantation procedures were performed by an experienced colorectal surgery consultant—(SR).

### Surgical procedure

**Fig. 1:**
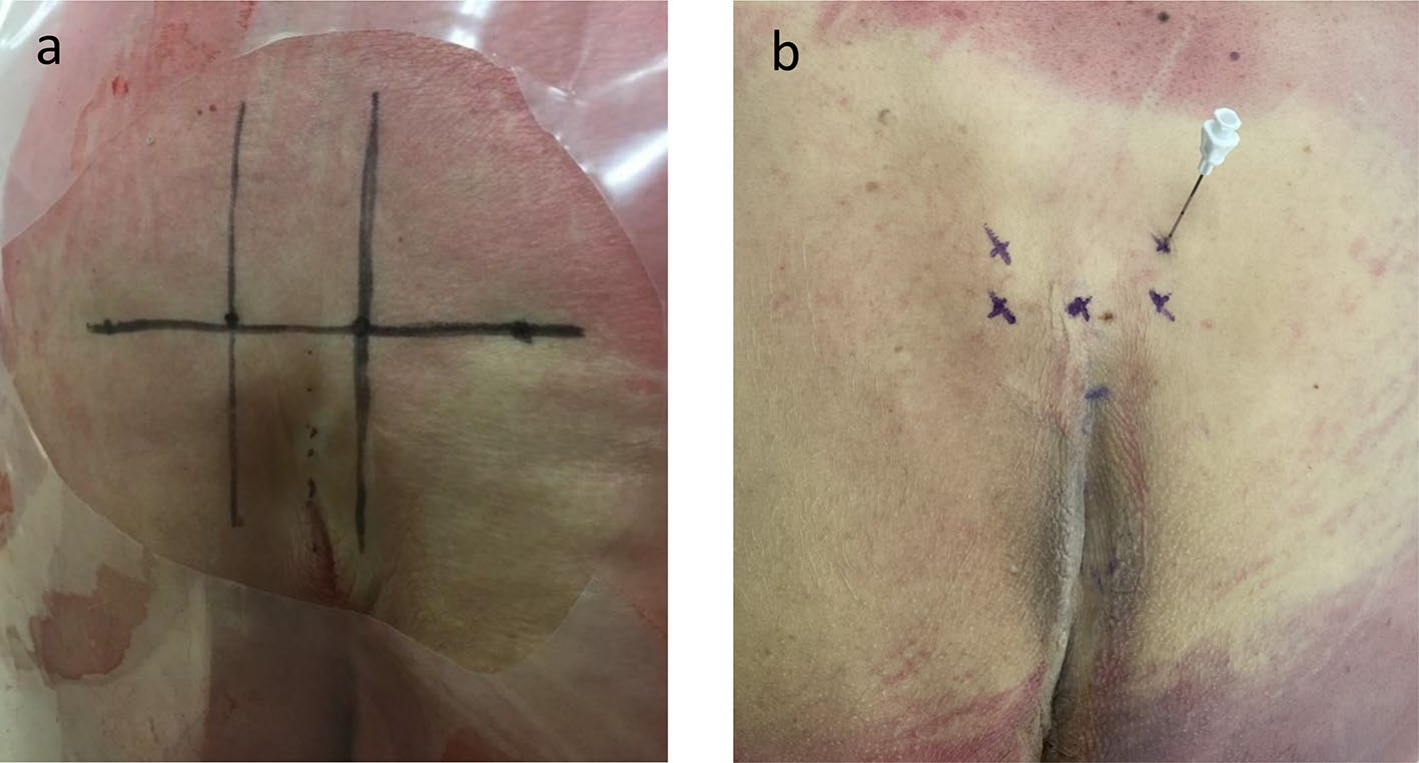
S3-marking **a** “H”-implantation technique and **b** conventional lead implantation, with the patient placed in prone position

In group A, SNM implantation was performed on full-body specimens. Cadavers were placed in a prone position. The lumbar lordosis was corrected. For percutaneous lead placement, the anatomical landmarks and borders of the sacrum were identified for conventional implantation as described by Williams et al. [12] and Deveneau et al. [9]: the needle was placed 9 cm superior to the tip of the coccyx, then one 2 cm lateral to the midline on the left and one 2 cm lateral to the midline on the right (Fig. 1). The needle was introduced until the sacral foramen was entered. Afterwards, the stylet of the foramen needle was removed and replaced with the guidewire. The tined lead was placed through the introducer sheath until all electrodes (E0–3) entered the foramen. At this point, latero-lateral and anterior–posterior fluoroscopy was carried out to confirm the exact location of the lead.

During the procedure, the distance between the intersection points and the insertion points, as well as the angle of the needle to the skin, were measured with a ruler and goniometer. In addition, the angle of the lead electrode and the sacrum was measured with a goniometer on the X-ray images.

### Dissection

After electrode placement, the pelvis was dissected to determine the exact location of the lead.

Initially, the cadavers were bisected between L3 and L4 with dissection and ligation of the sigmoid colon. The rectum was mobilized and resected, performing a total mesorectal excision (TME) to preserve the presacral fascia. Subsequently, the fascia was sharply dissected from cranio-medial to caudo-lateral to expose the nerves without displacing the leads. Finally, the nerves and the exact location of the leads were noted and documented using photographs with a digital reflex camera (CanonOES 5D Mark I, Canon Inc., Tokyo, Japan). The distances of the different electrodes (E0–3) to the nerves and the sacral foramina were measured.

Finally, the soft tissue and periosteum of the sacrum were removed to visualize the anterior and posterior surface of the sacral foramina and determine which sacral foramen was used for lead implantation.

The conventional implantation technique (group A) results were compared with those of the "H"-implantation technique (group B).

### Outcome measurements

The distance of the tined lead to the sacral nerve was assessed and compared between two defined implantation techniques. Secondary outcome parameters included differences in surgical complications and anatomical landmarks.

In addition, the insertion angle of the lead and the distance from marking to actual placement between the two comparative groups were analyzed.

### Statistical analysis

Continuous variables are expressed as median and interquartile ranges. Categorical variables are presented as numbers with percentages. To analyze primary and secondary endpoints, measurements were compared for the two implantation techniques. Quantitative variables were compared using the dependent Student’s *t*-test or the Wilcoxon test, as appropriate. To explore dichotomous variables, the “Chi-square test” was used. A *p*-value < 0.05 was considered significant. Statistical analysis was performed using the SPSS statistical software package (IBM SPSS Statistics for Mac, Version 22.0).

### Ethical approval

The local ethics committee approved this study (EK #2219/2018).

### Study registration

This study was registered at ClinicalTrials.gov (NCT04726904).

## Results

### Anatomical characteristics

The median posterior sacral foramen diameter measured 5 × 3 mm (2.5–7.0 mm × 2.0–7.0 mm) in group A and 5 × 7 mm (3.5–16.0 mm × 4.0–13.0 mm) (height *p* = 1.0; width *p* = 0.7) in group B.

In group A, the exit point of the nerve from the anterior sacral foramen was at the medial edge in 70% of the cases (10%—lateral; mediocranial—10%, mediocaudal—10%), in contrast to group B, where the nerve left the foramen always at the medial upper edge.

**Fig. 2:**
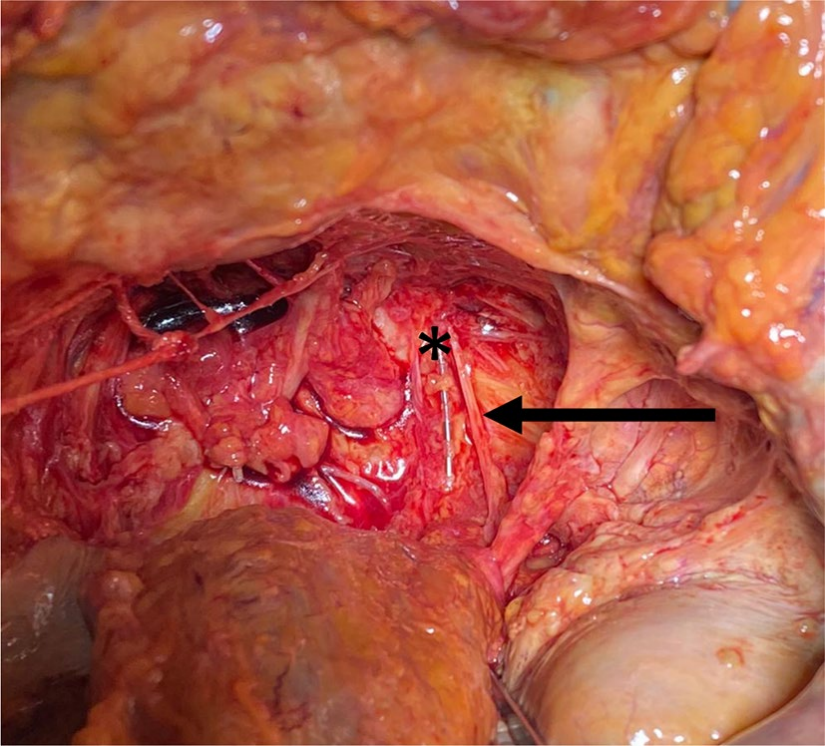
Cadaver group A (conventional implantation). The lead is positioned medial to the nerve as demonstrated after dissection with mobilization of the rectum and removal of the presacral fascia.* lead; *bolt* nerve

There was no significant difference in the size of the foramen according to sex (Group A height: 5.5 mm, Group B height: 5 mm, *p* = 0.91; Group A width: 6 mm, Group B width: 7 mm, *p* = 0.51) and side (Group A height: 5 mm, Group B height: 5 mm, *p* = 0.29; Group A width: 7 mm, Group B width: 7 mm, *p* = 0.26). In group A, there was no difference between the same aspects (sex: height: 4.6 mm (male) vs. 5.0 mm (female), *p* = 0.75; width: 4.8 mm (male) vs. 2.8 mm (female), *p* = 0.17; side: height, 4.8 mm (left) vs. 4.8 mm (right), *p* = 0.93; width, 3.8 mm (left) vs. 3.3 mm (right), *p* = 0.676). (Fig. 2).

### Electrode position

The skin was entered at a median angle of 58.5° (46–65°) in group A, and a median angle of 60° (50–65°) in group B. These data were not significantly different (*p* = 0.94).

As regards the distance of the electrodes (0–3) from the nerve root, there was no significant difference between the groups (E0 *p* = 0.969; E1 *p* = 0.754; E2 *p* = 1.000; E3 *p* = 0.675). Results are given in Table 1.

**Table 1:** Detailed distance of the leads in both groups

Electrode	Group A	Group B	*P *
	Median (range)	Median (range)	
Electrode 0 (E0)	0.0 (0–51)	0.0 (0–3)	0.969
Electrode 1 (E1)	0.0 (0–52)	0.5 (0–5)	0.754
Electrode 2 (E2)	2.5 (0–53)	2.5 (0–11)	1.000
Electrode 3 (E3)	3.5 (0–54)	4.0 (0–16)	0.675

Distance shown in mm electrode contact 3 (E3)—the most distal—to electrode contact 0 (E0)—the most proximal

**Fig. 3:**
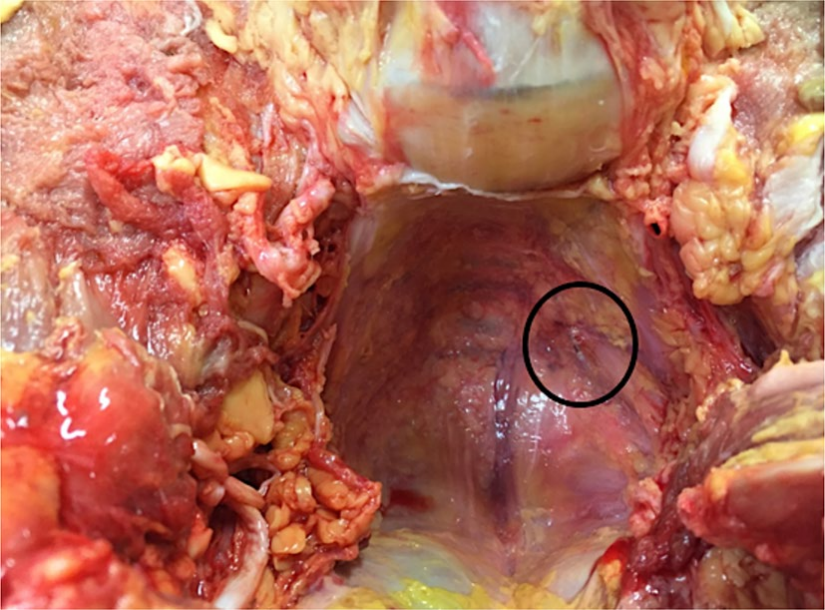
Cadaver group B (“H”-implantation technique), The lead has perforated the presacral fascia on the left sided neural foramen. The rectum has been mobilized

In addition, the presacral fascia was perforated in 3 lead placements (group A: *n* = 1 (12.5%), group B: *n* = 2 (20%). (see Fig. 3) In 2 cases (20%) of the conventional group A, the lead was misplaced and located at the gluteal muscle. Notably, neither entered the S3 foramen.

The position of the lead with respect to the nerve and the anterior sacral neuroforamen are outlined in Table 2.

**Table 2:** Detailed position of the nerve in both groups

Nerve to	Group A	Group B
	position	position
Lead		
Medial, *n* (%)	2 (25)	2 (20)
Lateral, *n* (%)	4 (50)	–
Cranial, *n* (%)	–	5 (50)
Caudal, *n* (%)	2 (25)	2 (20)
Perforated, *n* (%)	–	1 (10)
Neural foramen		
Medial, *n* (%)	8 (80)	1 (10)
Lateral, *n* (%)	1 (10)	5 (50)
Caudal, *n* (%)	1 (10)	4 (40)

Position of the nerve in relation to the lead and the neural foramen leaving the anterior sacral neural foramen

### Radiological outcome

Using anterior–posterior X-ray imaging, we found four (group A: *n* = 2 (10%), group B: *n* = 2 (10%) tips of the lead pointing straight, whereas the remaining 16 leads deflected to the lateral side. Even the leads that had a false placement were located on the anterior–posterior X-ray in a lateral pointing position.

## Discussion

Optimal lead placement is crucial for the functional outcome in SNM. The lead should be located close to the targeted sacral nerve. This may allow a low amplitude stimulation and enhanced programming. Notably, little is known about the ideal implantation technique. An expert group recently recommended a standardized approach using fluoroscopy and “H” marking to deliver the best patient outcome in fecal incontinence. Although not supported by the results of clinical studies, Matzel et al. provided a description of their implantation technique. This expert group proposed this new implantation technique will optimize treatment response by ensuring the best lead placement close to the nerve [13]. In addition, the standardized approach allows a better understanding of anatomical landmarks and aids in training.

It is unclear whether the “H” technique results in closer proximity of the tined lead to the sacral nerve compared with other implantation techniques. Müller et al. recently suggested that close contact between the nerve and the electrode could be achieved. The authors dissected five pelvic cadavers after SNM implantation, demonstrating a median distance of 0 mm (0–3 mm) for the most proximal electrode to 1.75 mm (0–16 mm) for the most distant electrode to the nerve. However, no comparison to other implantation techniques was made.

The present study is the first cadaver study comparing two different implantation techniques. For the comparator, we used the commonly performed technique described by Williams et al. [12] and Deveneau et al. [9].

Both techniques were performed by an experienced colorectal surgery consultant—SR. Surprisingly, no difference regarding the distance of the lead from the nerve was observed. One may speculate that this was due to the experience of the surgeon and that a less experienced SNM surgeon would have better results when performing the more standardized “H”-guided approach.

Only a few anatomical studies have focused on the optimal insertion technique and specific landmarks [9, 14, 15]. Buchs et al. analysed insertion angles [14]. The authors inserted SNM electrodes into the S3 foramina in five cadavers using the same conventional technique as this study. The lead placement was recorded with a laparoscopic camera controlling the position of the needle and electrode in relation to the nerve. Before implantation, the cadaver was dissected to visualize the presacral fascia and the underlying nerves. The mean angle of insertion in the sagittal plane was 62.9 ± 3° (59–70). In the axial plane, the mean angle for the left side was 91.7 ± 13.5° (80–110) and 83.2 ± 7.7° for the right side (75–95). The authors concluded that these angles resulted in optimal placement of the leads along the S3 sacral root but did not record the distance from the lead to the nerve [14].

Conversely, Deveneau et al. described landmarks for implantation and compared them to the standard 9 cm from the tip of the coccyx—S3 implantation technique [9]. They found a distance of 9 cm from the tip of the coccyx and 2 cm lateral to the midline was a reasonable starting landmark for percutaneous nerve evaluation. They commented that if a clear response is not obtained, either a complete stage one implantation or the use of fluoroscopy is required.

Hasan et al., again using a cadaveric approach, compared implantation of the electrodes in all sacral foramina and described potential complications regarding the proximity to anatomical structures [15]. They observed a higher incidence of nerve root penetration with the S1 foramen needle insertion. In addition, they highlighted that the S3 and S4 nerve roots are surrounded by venous plexuses and therefore represent a potential source of venous haemorrhage [15].

Our study found eight leads entering S3 in group A, with two misplaced leads due to exophytes. In contrast, we found all ten leads entering the S3 in group B. This supports the concept of fluoroscopy-guided lead implantation with clear anatomical landmarks and a lower likelihood of false tracts.

We observed a variety of electrode positions relative to anatomical structures in both groups. Perforation of the nerve and a cranial course of the nerve with respect to the lead were only seen in group B. Conversely, a lateral course of the nerve with respect to the lead was only seen in group A.

There is a paucity of data regarding the lead position in clinical studies. In particular, little is known as to whether lead position relative to the nerve affects clinical outcome [16]. The type of implantation varies, with no standardized technique defined in previous trials. This is surprising given the longevity of SNM surgery. A recent systematic review observed no consensus over predictive factors for success in SNM [16]. It included only two studies that examined the effect of electrode position, determined by the depth, angle and deflection with respect to the sacral foramen. No specific parameter was associated with a more successful outcome [17]. The proximity of the electrode to the nerve was not evaluated in these trials. Adelstein et al. reported on the optimization of lead implants through the use of a curved stylet and precise knowledge of the S3 nerve anatomy [18].

The limitations of the present study include the cadaveric design and the obvious lack of functional outcomes. The use of muscular stimulation to determine effective results is an essential element of lead placement and is clearly not possible in cadavers. However, this is the first study evaluating different surgical SNM techniques and their impact on the proximity of the lead to the targeted nerve. One can speculate that this does have a significant influence on the latter clinical success rate.

## Conclusions

Using cadavers, we have demonstrated that the fluoroscopy-guided “H” technique did not result in a closer contact of the lead to the sacral nerve compared with the commonly utilized implantation approach defined by bony landmarks. However, lead misplacement was less likely with the “H” technique.
